# Inflexible Orbitofrontal Cortex Functional Connectivity From Rest to Acute Stress in Alcohol Use Disorder

**DOI:** 10.1111/adb.70083

**Published:** 2025-08-17

**Authors:** Dylan E. Kirsch, Tiffany C. Ho, Kate M. Wassum, Lara A. Ray, Erica N. Grodin

**Affiliations:** ^1^ Department of Psychology University of California, Los Angeles Los Angeles California USA; ^2^ Department of Psychiatry and Biobehavioral Sciences University of California, Los Angeles Los Angeles California USA

**Keywords:** addiction, alcohol, amygdala, hippocampus, prefrontal cortex, resting‐state fMRI, stress

## Abstract

Adaptive stress coping is often impaired in individuals with alcohol use disorder (AUD). This process relies on neurocircuitry involved in emotional and behavioural regulation, particularly the ventromedial PFC (vmPFC) and orbitofrontal cortex (OFC), along with limbic and ventral striatal regions (e.g., amygdala, hippocampus and nucleus accumbens). These systems are highly sensitive to the neurotoxic effects of alcohol, which may disrupt their ability to flexibly adapt in response to acute stress. This study investigated state‐dependent changes (termed ‘flexibility’) in vmPFC‐limbic/striatal and OFC‐limbic/striatal functional connectivity from rest to acute stress in individuals with AUD versus matched controls and examined associations with coping strategies. Twenty‐four adults with AUD (*age*_*mean*_ = 33, 11F) and 23 matched controls (*age*_*mean*_ = 32, 11F) underwent fMRI during resting‐state followed by the Montreal Imaging Stress Task (MIST) and completed the COPE Inventory. Functional connectivity between vmPFC‐limbic/striatal and OFC‐limbic/striatal regions was assessed during rest and stress (MIST) conditions. Group differences in state‐dependent changes in functional connectivity were analysed using repeated‐measures ANCOVA. Functional connectivity between the right OFC–right amygdala and right OFC–right hippocampus increased from resting‐state to the MIST in the control group, but this shift was not present in the AUD group (group x condition, *p*_*FDR*_ < 0.05). Although connectivity did not differ between groups during the MIST (*p*'s > 0.2), the AUD group exhibited elevated connectivity between these regions at rest (*p*'s < 0.05). Moreover, among controls, increased right OFC–right hippocampus connectivity from rest to MIST was associated with more adaptive versus maladaptive coping (*p* < 0.05). Compared to controls, individuals with AUD exhibited a pattern of inflexible OFC‐amygdala and OFC‐hippocampus functional connectivity under changing stress conditions. Diminished stress‐related connectivity changes in AUD appeared to be driven by elevated functional connectivity at rest. Future studies should test whether this resting‐state connectivity pattern reflects an allostatic state that constrains the system's capacity to flexibly respond to acute stress.

## Graphical abstract

Individuals with alcohol use disorder (AUD) showed reduced flexibility in orbitofrontal cortex (OFC) connectivity with the amygdala and hippocampus when shifting from rest to acute stress states, compared to matched controls. This inflexibility was driven by elevated resting‐state connectivity in AUD, rather than differences during stress. In controls, greater OFC‐hippocampus connectivity increases from resting‐state to stress were linked to more adaptive versus maladaptive coping, suggesting that inflexible functional connectivity in AUD may limit the ability to employ effective coping strategies.

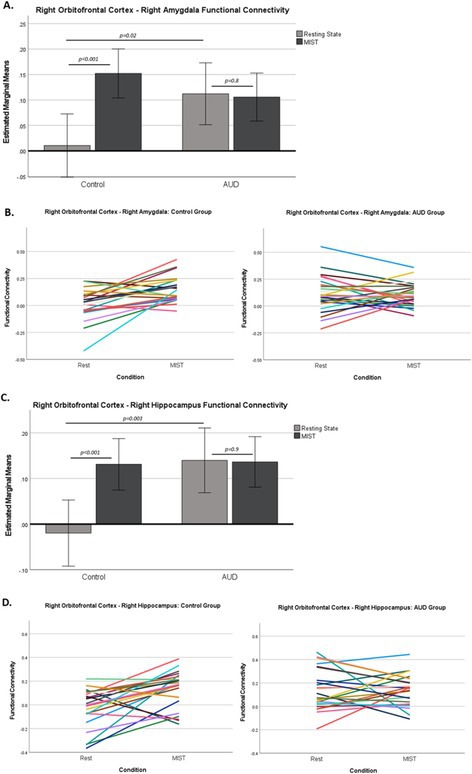

## 1 Introduction

Stress plays a critical role in the onset and maintenance of alcohol use disorder (AUD), serving as a common motivator for alcohol use and trigger of relapse [1, 2, 3, 4, 5]. Theoretical models (i.e., social learning theory and social cognitive models of relapse) and empirical studies posit that some individuals with AUD may use alcohol to cope with stress due to a lack of more adaptive stress coping skills (e.g., active coping and seeking social support) [6, 7, 8, 9, 10, 11]. Adaptive stress coping relies on the brain circuitry involved in emotional and behavioural control [12]. The ventral prefrontal cortex (vPFC)—encompassing ventromedial PFC (vmPFC) and orbitofrontal cortex (OFC)—is a key locus of a resilient stress coping system [12, 13, 14]. It is highly sensitive to the neurotoxic effects of alcohol [14, 15, 16, 17]. The vPFC shares structural and functional connections with stress‐responsive limbic and ventral striatal structures, including the amygdala, hippocampus and nucleus accumbens [15, 18]. Together, these regions play an integrated role in coordinating behaviours in service of an adaptive stress response [12, 13, 14, 19, 20, 21, 22].

Both preclinical and human literature support an association between chronic alcohol exposure and maladaptive changes in vPFC and limbic/striatal regions [23, 24, 25]. Preclinical research has demonstrated a causal link between excessive alcohol consumption and decreased neuronal and glial cell survival in the vPFC, amygdala and hippocampus, as well as in the white matter tracts connecting these regions [26, 27]. Human neuroimaging studies have reported chronic alcohol‐related alterations in vPFC system function, both during acute stress and at rest [28, 29]. Specifically, individuals with AUD exhibit vPFC hypoactivity in response to acute stress but hyperactivity under neutral conditions [30, 31]. This response pattern has been linked to a shorter time to relapse, more frequent alcohol use and heightened stress‐induced craving [30, 31]. Additionally, alcohol‐related alterations in resting‐state functional connectivity have been observed in vPFC networks, with studies finding elevated OFC‐ventral striatal connectivity in individuals with AUD and those who binge drink [32, 33]. A recent systematic review found that individuals with AUD and those who binge drink exhibit stronger positive functional connectivity between the PFC and limbic structures (e.g., amygdala) compared to controls [28], a pattern of connectivity that has been associated with a sustained stress state (i.e., elevated cortisol) in healthy men [34]. Collectively, these findings suggest that chronic alcohol‐related neuroadaptations in the vPFC and in its connections to limbic and striatal regions may contribute to an impaired adaptive stress response, ultimately promoting continued alcohol use.

Disruptions in these vPFC stress response systems in individuals with AUD during both rest and acute stress states could suggest impaired neural flexibility under changing stress conditions. The concept of state‐dependent reconfiguration or flexibility of functional networks has gained increased attention in neuroimaging research over the past decade [35, 36, 37, 38, 39, 40]. This phenomenon has often been investigated by comparing functional connectivity patterns between resting‐state and task‐evoked conditions, with greater shifts in functional connectivity reflecting greater neural flexibility [35, 36, 37, 38, 39, 40]. A growing body of literature supports that such flexible reconfiguration of brain networks as a function of state (i.e., rest versus task) supports adaptive cognitive control [37, 41, 42]. This framework has been applied to understanding the neural underpinnings of psychopathology, with studies identifying neural inflexibility—characterized by greater similarity in resting‐state and task‐based functional connectivity—in individuals with mood disorders [36, 43].

Therefore, examining state‐dependent changes in functional connectivity, specifically shifts from rest to acute stress conditions, provides a valuable framework for understanding neural flexibility under changing stress states in AUD. In this study, we applied this approach to test the hypothesis that neural flexibility of vPFC‐limbic/striatal connectivity is diminished in adults with AUD compared with healthy controls and that diminished flexibility is associated with less adaptive and more maladaptive stress coping strategies. Adults with AUD and matched controls underwent resting‐state fMRI immediately followed by the Montreal Imaging Stress Task (MIST), a well‐established paradigm that evokes acute stress responses [44]. We assessed functional connectivity changes between vmPFC and OFC with subcortical limbic and ventral striatal regions (amygdala, hippocampus and nucleus accumbens) under both rest and stress conditions. We predicted that (1) individuals with AUD would exhibit reduced neural flexibility, defined by limited state‐dependent shifts in vmPFC‐limbic/striatal and OFC‐limbic/striatal connectivity from rest to stress, compared to healthy controls and (2) diminished neural flexibility in AUD would be associated with less adaptive and more maladaptive coping strategies.

## 2 Methods

### 2.1 Data Source and Sample

This is a secondary analysis of data collected for a parent study examining the effects of stress on cognition in individuals with and without AUD (R21AĂ43). Participants were recruited from the greater Los Angeles metropolitan area between June 2023 and August 2024 through online and newspaper advertisements, campaigns in mass transit and targeted recruitment through a UCLA Addiction's Laboratory database of previous study participants. All study procedures were approved by the UCLA Institutional Review Board. All participants provided written informed consent following a full explanation of study procedures. Seventy‐three participants were enrolled in the parent study. Of those, 47 participants (*N* = 24 with AUD; *N* = 23 controls) completed both resting‐state and MIST scans and therefore comprised the sample for this secondary study. All participants who completed the scans provided usable data.

As part of enrollment criteria for the parent study, participants were between the ages of 18 and 65. Eligibility criteria for participants in the AUD group included (1) current (past 3 months) moderate or severe AUD as defined by DSM‐5 criteria; (2) heavy drinking levels, as defined by the NIAAA as ≥ 14 drinks/week for men and ≥ 7 drinks/week for women; and (3) Alcohol Use Disorder Identification Test (AUDIT) score ≥ 8. Eligibility criteria for participants in the control group included (1) no lifetime moderate or severe AUD; (2) no mild, moderate or severe AUD over the past 5 years; and (3) AUDIT score < 7. Exclusion criteria for participants in both groups included (1) current treatment for alcohol use or history of treatment in the 30 days before enrollment or treatment seeking; (2) current (past 12 months) DSM‐5 diagnosis of substance use disorder (SUD) for any psychoactive substances other than nicotine; (3) lifetime DSM‐5 diagnosis of schizophrenia, bipolar disorder or any psychotic disorder; (4) current DSM‐5 major depressive disorder (MDD) with suicidal ideation; (5) current use of psychoactive drug, as determined by urine toxicology screen, except for tetrahydrocannabinol (THC); (6) pregnancy, nursing or refusal to use a reliable method of birth control if female; (7) nonremovable ferromagnetic objects in the body or any other contraindication to MRI; (8) claustrophobia; and (9) lifetime history of serious head injury or prolonged period of unconsciousness (> 30 min).

### 2.2 Study Procedures

Following initial telephone screening, participants were invited to the laboratory for a screening visit, during which they completed a battery of assessments for eligibility and measures on adaptive coping, recent substance use and demographics. Eligible participants returned to the laboratory for an fMRI scan, which included a structural scan for registration and then a resting‐state scan, followed by the MIST scan. Participants were required to have a breath alcohol concentration of 0.00 g/dL, Clinical Institute Withdrawal Assessment for Alcohol Scale Revised (CIWA‐Ar) [45] score < 10 at each in‐person visit and test negative for all psychoactive drugs, as determined by urine toxicology screen. Individuals who tested positive for THC, however, were eligible to complete study visits.

### 2.3 Assessments

Clinical and demographic characteristics were measured using clinician‐administered and self‐report assessments. The Structured Clinical Interview for DSM‐5 (SCID‐5) [46] was used to assess for AUD, other current SUDs and current MDD. The SCID was also used to assess for lifetime DSM‐5 diagnosis of schizophrenia, bipolar disorder or any psychotic disorder, which were exclusion criteria. The Timeline Followback (TLFB) Interview [47] was used to assess alcohol, cigarette and cannabis use in the 30 days prior to enrollment. Using this assessment, we calculated total drinks, total drinking days, drinks per drinking day, cannabis use days and total cigarettes smoked (among those who reported past month cannabis/cigarette use) over the past 30 days. The Fagerstrom Test for Nicotine Dependence (FTND) was administered to determine cigarette smoking status (i.e., never, occasional or daily smoking) [48]. A standard demographics form was used to assess age, sex at birth, race and ethnicity. Participant demographic and clinical characteristics are detailed in Table 1.

**TABLE 1:** Sample characteristics.

		Control	AUD	*p* value
		*N* = 23	*N* = 24	
Demographics				
Age (± SD)		31.6 ± 12.5	32.5 ± 9.1	0.8
Male (%)		12 (52)	13 (54)	0.9
Female (%)		11 (48)	11 (46)	
Race	White (%)	11 (48)	7 (29)	0.4
	Black or African American (%)	2 (9)	5 (21)	
	Asian (%)	5 (22)	4 (17)	
	American Indian or Alaska Native (%)	0	1 (4)	
	Mixed race (%)	2 (9)	3 (13)	
	Other (%)	3 (13)	4 (17)	
Ethnicity	Hispanic/Latino (%)	5 (22)	8 (3)	0.4
Alcohol use characteristics				
Current AUD moderate (%)		—	12 (50)	—
Current AUD severe (%)		—	12 (50)	
TLFB drinking days (past 30 days) (± SD)		5.2 ± 5.3	17.4 ± 7.8	**< 0.001**
TLFB total drinks (past 30 days) (± SD)		10.0 ± 11.9	105.0 ± 69.0	**< 0.001**
TLFB drinks per drinking day (past 30 days) (± SD)		2.0 ± 0.9	6.5 ± 4.1	**< 0.001**
Cigarette and cannabis use characteristics				
Cigarette smoking (%)	Not at all	22 (96)	18 (75)	0.1
	Occasional	1 (4)	4 (17)	
	Daily	0	2 (8)	
TLFB total cigarettes (past 30 days) (± SD)^a^		33	80.3 ± 112.3	0.7
Current cannabis use disorder (%)		0	4 (17)	0.1
# reporting past month cannabis use (Y/N; %)		5 (22)	7 (29)	0.6
TLFB cannabis use days (past 30 days) (± SD)^a^		6.0 ± 9.5	8.1 ± 5.7	0.6
Positive toxicology screen—THC (%)^b^		1	4 (17)	0.2
Other psychiatric characteristics				
Current major depressive disorder (%)		0	4 (17)	0.1
Coping strategies				
Adaptive coping (± SD)		89.6 ± 10.7	86.2 ± 10.8	0.4
Avoidant coping (including substance use subscale) (± SD)		25.4 ± 6.8	34.8 ± 8.5	**< 0.001**
Avoidant Coping (without substance use subscale) (± SD)		20.5 ± 5.4	24.6 ± 5.9	**0.02**

*Note:* Bold text indicates significant differences between groups.

Abbreviations: AUD, alcohol use disorder; THC, tetrahydrocannabinol; TLFB, timeline followback.

^a^ Among participants who report use over the past 30 days.

^b^ Toxicology screen on day of fMRI scan.

Participants completed the Spielberger State–Trait Anxiety Inventory‐6 (STAI‐6) [49] and the Subjective Units of Distress Scale (SUDS) [50] immediately before and after to assess the effect of stress, via the MIST, on anxiety and distress, respectively.

Coping strategies were measured using the 60‐item COPE Inventory [51]. Participants reported what they generally do when they experience stress on a 4‐point scale, ranging from 1 (*I usually don't do this at all*) to 4 (*I usually do this a lot*). The COPE has 15 subscales (each comprised of four items), each of which reflect a specific coping strategy. Based on prior work [10], we combined scores from subscales that have most commonly been identified as ‘adaptive’ or ‘useful’ coping methods [51, 52, 53] to obtain a composite ‘adaptive coping’ score. These subscales included active coping, planning, suppression of competing activities, restraint coping, positive reinterpretation and growth, religion, acceptance, seeking social support—instrumental—and seeking social support—emotional. To assess maladaptive coping strategies, we generated a composite ‘avoidant coping’ score by combining: behavioural disengagement, denial, mental disengagement and substance use subscales. Given that study groups were recruited based on their alcohol use, we generated a second composite ‘avoidant coping’ score that did not include the substance use subscale to reduce the possibility of criterion contamination. Coping scores are detailed in Table 1.

### 2.4 Montreal Imaging Stress Task

A modified version of the MIST was administered to induce acute stress during fMRI. This modified task was previously validated in adults with moderate‐to‐severe AUD [54]. The task was modified from its initial implementation [44] to include more runs. As previously described [54], participants completed a training session prior to their MRI scan to familiarize themselves with the task and to assess the participants' ability to perform mental arithmetic (i.e., average amount of time needed to solve problems at four difficulty levels).

Participants then completed three runs of the MIST during fMRI. Each run was ~7 min, yielding a total task time of ~21 min. Each run was comprised of two stress (90‐s), control (90‐s), and rest (30‐s) blocks. Blocks were pseudo‐randomly presented within each run. During the stress and control blocks, participants were presented with arithmetic problems and participants selected the numerical solution on a rotary dial using a button box. Participants received feedback (‘correct’ or ‘incorrect’) on their response before the next trial began. During the stress blocks, the programme was initially set to a time limit that was 10% less than the average response time derived from the training session. During the stress block, the programme then dynamically modulated timing, such that if the participant had three consecutive correct responses, the programme reduced the time limit to 10% less than the average time for the three correct problems. Conversely, if the participant had three consecutive incorrect responses, the programme increased the time limit by 10%. Also during the stress condition, participants were presented with a colour bar at the top of the screen, which served as a performance indicator. The bar presented the participant with their own performance and the ‘average’ performance of all participants, which was artificially set at 10%–90% accuracy (see [54]). During the control blocks, participants were presented with arithmetic problems that were of similar difficulty to those presented during the experimental condition; however, there were no preset time limits or performance indicators. Participants were still instructed to complete the arithmetic problems as quickly and accurately as possible. Finally, the rest blocks included the presentation of the task interface without any arithmetic problems. Between runs, participants were given negative verbal feedback about their task performance and were told they needed to improve their performance in order for their data to be used in the study. Following completion of the scan and collection of subjective anxiety and distress ratings, participants were debriefed about the MIST and given the opportunity to ask questions.

### 2.5 MRI Data Acquisition

Neuroimaging took place on a 3.0T Siemens Magneton Prisma scanner (32‐channel head coil) at the UCLA Center for Cognitive Neuroscience (CCN). A T1‐weighted MPRAGE sequence (TR/TE/TI = 1900/2.26/900 ms, flip angle = 9°, voxel size = 1.0 mm^3^, FOV = 250 mm, ~6:50 min) was acquired for coregistration of fMRI data. During the resting‐state scan, blood oxygenation level‐dependent (BOLD) signal was measured with a T2* gradient‐echo‐planar image (EPI) sequence (TR/TE = 800/37 ms, voxel size = 2.0 mm^3^, slices = 72 axial slices, slice thickness = 2.0 mm, FOV = 208 mm, 6:40 min). Participants were presented with a cross hair on a screen. They were instructed to stay awake with their eyes open and to focus on the cross hair. BOLD signal during the MIST was measured with three T2*‐weighted EPI sequences, using identical parameters to the resting‐state scan, except for the length of scan, which was approximately 7 min.

### 2.6 MRI Preprocessing and First‐Level Analysis

Structural and functional image preprocessing was completed using fMRIPrep 23.1.4 (RRID:SCR_016216) [55], in line with prior work [56]. Functional preprocessing included generating a reference volume and its skull‐stripped version. Following this, the preprocessing pipeline included motion correction and nuisance estimation. BOLD reference images were coregistered to the T1w reference [57] with the boundary‐based registration cost function [58]. The BOLD time series were resampled into standard space, generating a preprocessed BOLD run in MNI152NLin2009cAsym space. Motion outliers were identified for frames that exceeded a threshold of 0.5‐mm framewise displacement or 1.5 standardized DVARS. No participants were excluded from this analysis for exceeding these thresholds.

Data were imported into the CONN toolbox (www.nitrc.org/projects/conn) [59] for SPM12 further processing and analyses. Preprocessing steps in CONN included smoothing and denoising. Smoothing was completed using a 4‐mm FWHM Hamming filter (two times the voxel size). Denoising included aCompCor (anatomical component analysis correction) regression [37], followed by quadratic detrending and low band‐pass filtering (0.008–0.09 Hz). Nuisance regressors included cerebrospinal fluid and white matter, six motion parameters and their first derivative, scrubbing parameters (all motion outliers) and session effects. In alignment with prior studies examining neural flexibility or reconfiguration, preprocessing steps were identical for resting‐state and MIST fMRI data [35, 36, 43, 60].

Using the CONN Toolbox, we performed a ROI‐to‐ROI bivariate correlation first‐level analysis to calculate Fisher‐transformed correlation coefficients (*z* scores) between a priori ROI‐to‐ROI connections during resting‐state and the MIST (entire scan, independent of specific blocks [35, 36, 60]). We extracted functional connectivity values (*z* scores) between ROI‐to‐ROI connections during rest and MIST for further statistical analyses (see the next section). A priori ROIs were defined using the FSL Harvard Oxford Atlas in the CONN toolbox and included vmPFC, right and left OFC, right and left OFC amygdala, right and left OFC hippocampus and right and left nucleus accumbens. We examined ROI‐to‐ROI connections between vPFC regions (vmPFC and OFC) with subcortical regions (amygdala, hippocampus and nucleus accumbens) and only examined unilateral connections (i.e., right OFC to right amygdala), resulting in a total of 12 ROI‐to‐ROI pairs.

### 2.7 Statistical Analysis

Analyses were conducted using IBM SPSS Statistical Software Version 28. We examined between‐group differences in demographic and clinical characteristics. Between‐group differences in continuous variables were assessed using a *t* test or Wilcoxon test, as appropriate, and between‐group differences in categorical variables were assessed using a chi‐square or Fisher's exact test, as appropriate. To assess the effects of the MIST on subjective feelings of anxiety (STAI) and distress (SUDS), we conducted repeated measures ANCOVA models. Condition (pre‐fMRI vs. post‐fMRI) was the two‐level within‐subjects factor, group (control, AUD) was the between‐subject factor, STAI and SUDS were the repeated measure outcome variables (modelled separately) and age and biological sex were covariates. Significant results are reported below (*p* < 0.05).

We conducted repeated measures ANCOVA to test our primary aim—that is, examining changes in functional connectivity from resting‐state to MIST in AUD versus controls. Condition (resting‐state, MIST) was the two‐level within‐subject factor, group (control, AUD) was the between‐subject factor and ROI‐to‐ROI functional connectivity (each of the 12 connections modelled separately) was the repeated measure outcome variable. Age and biological sex were included as covariates. To account for multiple comparisons (i.e., 12 ROI‐to‐ROI comparisons), we implemented an FDR‐correction using the p.adjust function in R Statistical Software. Significant results (*p*_*FDR*_ < 0.05) are reported below. Then, to examine associations with coping strategies, we created a single composite coping score by calculating the difference in the total adaptive coping score minus the total avoidant coping score (excluding substance coping) for each participant. Using the PROCESS v4.2 (model 1) in SPSS, we tested whether the group moderated the relationship between change in functional connectivity from rest to MIST and coping, controlling for age and biological sex. We only examined ROI‐to‐ROI connections identified as significant in the ANCOVA models testing our primary aim. Results were considered significant at *p* < 0.05.

To further characterize functional connectivity findings, we conducted a series of post hoc exploratory and sensitivity analyses, limited to ROI‐to‐ROI connections that showed significant effects in the primary analyses. First, we examined whether changes in subjective anxiety (STAI) and distress (SUDS) were associated with changes in functional connectivity by computing partial correlations within each group, controlling for age and biological sex. Second, we tested for potential hemispheric laterization effects using three‐way ANOVA with hemisphere and condition as the within‐subject factors, group as a between‐subject factor and ROI‐to‐ROI functional connectivity as the dependent variable. Third, to evaluate potential confounding by signal quality, we computed voxel‐wise temporal signal‐to‐noise ratio (tSNR) within significant ROIs and compared values between groups using independent‐sample *t* tests. Finally, we conducted a sensitivity analysis excluding individuals who tested positive for THC prior to fMRI scan to determine whether the primary findings remained significant. These analyses were conducted post hoc and were intended to further characterize findings identified in the primary analysis; therefore, we did not correct for multiple comparisons.

## 3 Results

### 3.1 Sample Characteristics

As expected, the AUD group reported more past month alcohol use compared to the control group (drinking days, total drinks and drinks per drinking day; all *p*s < 0.001). Groups did not differ on other demographic, clinical or substance use variables (all *p*s > 0.1). Regarding coping strategies, the AUD group reported greater use of avoidant coping strategies, both when including (*p* < 0.001) and excluding the substance use subscale (*p* = 0.02). Groups did not differ in terms of adaptive coping strategies (*p* = 0.4).

### 3.2 Subjective Anxiety and Distress Ratings—MIST Stress Manipulation Check

Across both groups, participants reported greater anxiety (STAI; *F*_[1, 45]_ = 4.9, *p* = 0.03, *ηp* ^2^ = 0.1) and distress (SUDS; *F*_[1, 45]_ = 4.6, *p* = 0.04, *ηp* ^2^ = 0.1) after the MIST, compared to before. There was a main effect of group on distress (*F*_[1, 43]_ = 5.8, *p* = 0.02, *ηp* ^2^ = 0.1) such that the AUD group reported greater distress both before and after the MIST. There was no main effect of group on anxiety (*F*_[1, 43]_ = 2.5, *p* = 0.1, *ηp* ^2^ = 0.06). There was no MIST by group interaction on anxiety (*F*_[1, 45]_ = 0.04, *p* = 0.8, *ηp* ^2^ = 0.001) or distress (*F*_[1, 45]_ = 1.4, *p* = 0.3, *ηp* ^2^ = 0.03). See Figure S1.

### 3.3 Group Differences in Resting‐State Versus MIST Functional Connectivity

**FIGURE 1:**
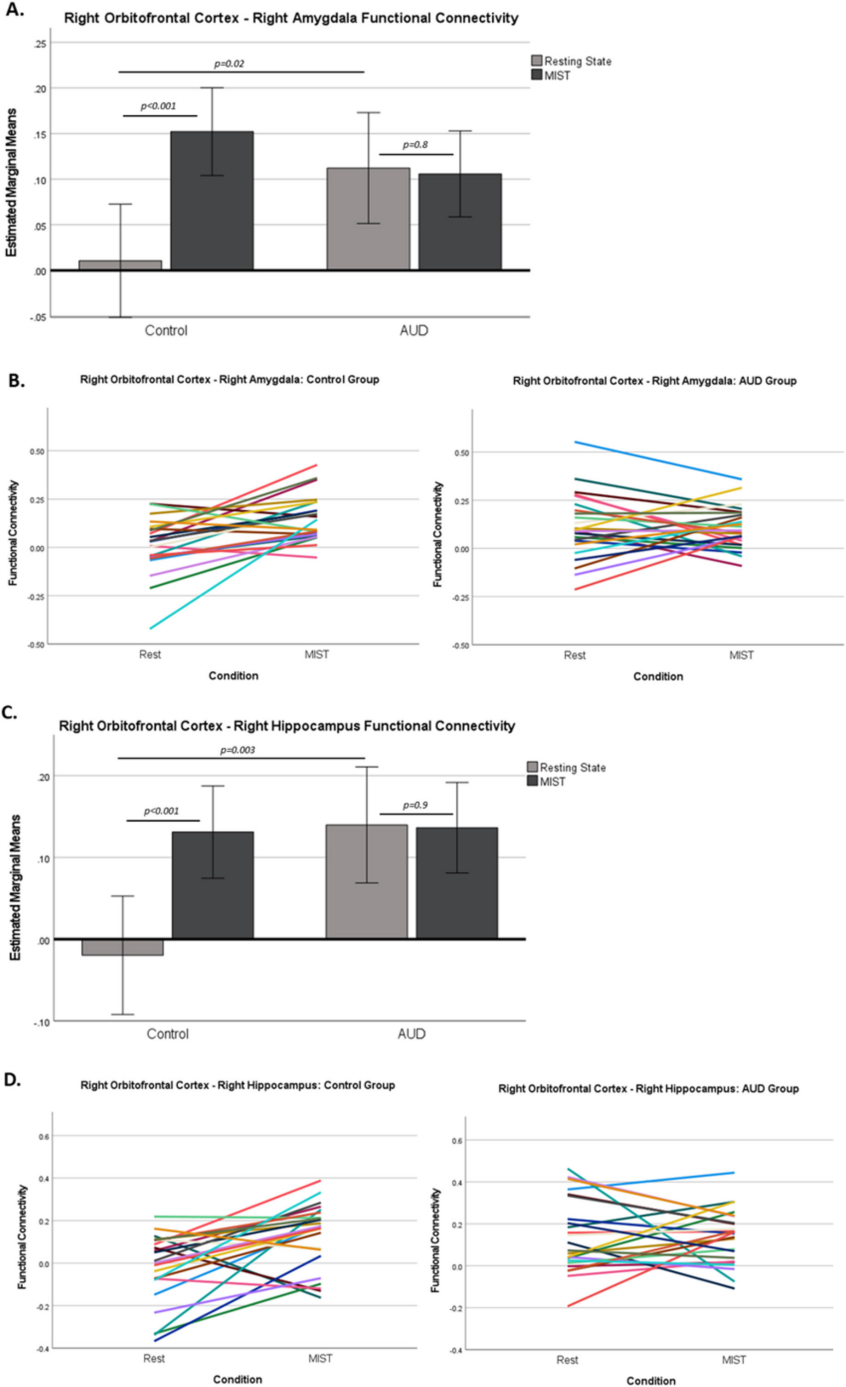
Functional connectivity between right orbitofrontal cortex (OFC)–right amygdala and right OFC–right hippocampus in the control and alcohol use disorder (AUD) groups during resting‐state and the Montreal Imaging Stress Task (MIST). (A, B) There was a group by condition interaction on right OFC–right amygdala functional connectivity (*p*_*FDR*_ = 0.02). The control group exhibited an increase in functional connectivity from resting‐state to MIST (*p* < 0.001), whereas the AUD group did not show a change in functional connectivity between conditions (*p* = 0.8). Additionally, the control group exhibited lower right OFC–right amygdala functional connectivity compared with the AUD group during resting‐state (*p* = 0.02), but groups did not differ in functional connectivity during the MIST (*p* = 0.2). Panel A displays average estimated marginal means for each group. Panel B displays raw connectivity values for each participant. (C, D) Similarly, there was a group by condition interaction on right OFC–right hippocampus functional connectivity (*p*_*FDR*_ = 0.048). The control group exhibited an increase in functional connectivity from resting‐state to MIST (*p* < 0.001), whereas the AUD group did not show a change in functional connectivity (*p* = 0.9). Additionally, the control group exhibited lower right OFC–right hippocampus functional connectivity compared with the AUD group during resting‐state (*p* = 0.003), but groups did not differ in functional connectivity during the MIST (*p* = 0.9). Panel C displays average estimated marginal means for each group. Panel D displays raw connectivity values for each participant. Error bars represent 95% confidence interval.

There was a group by condition interaction on right OFC–right amygdala functional connectivity (*F*_[1, 45]_ = 10.4, *p*_*FDR*_ = 0.02, *ηp* ^2^ = 0.20; Figure 1A,B). The control group exhibited an increase in functional connectivity from resting‐state to MIST (*F*_[1, 22]_ = 18.7, *p* < 0.001, *ηp* ^2^ = 0.3), whereas the AUD group did not show a change in functional connectivity between conditions (*F*_[1, 23]_ = 0.04, *p* = 0.8, *ηp* ^2^ = 0.001). Additionally, the control group exhibited lower right OFC–right amygdala functional connectivity compared with the AUD group during resting‐state (*F*_[1, 43]_ = 5.6, *p* = 0.02, *ηp* ^2^ = 0.1), but groups did not differ in functional connectivity during the MIST (*F*_[1, 43]_ = 1.2, *p* = 0.2, *ηp* ^2^ = 0.04). Similarly, there was a group by condition interaction on right OFC–right hippocampus functional connectivity (*F*_[1, 45]_ = 7.8, *p*_*FDR*_ = 0.048, *ηp* ^2^ = 0.15; Figure 1C,D). The control group exhibited an increase in functional connectivity from resting‐state to MIST (*F*_[1, 23]_ = 14.7, *p* < 0.001, *ηp* ^2^ = 0.3), whereas the AUD group did not show a change in functional connectivity (*F*_[1, 23]_ = 0.008, *p* = 0.9, *ηp* ^2^ = 0.0). Additionally, the control group exhibited lower right OFC–right hippocampus functional connectivity compared with the AUD group during resting‐state (*F*_[1, 43]_ = 10.03, *p* = 0.003, *ηp* ^2^ = 0.2), but groups did not differ in functional connectivity during the MIST (*F*_[1, 43]_ = 0.02, *p* = 0.9, *ηp* ^2^ = 0.0). All other ROI‐to‐ROI functional connectivity pairs were nonsignificant. There were no significant effects of age or biological sex. Table 2 shows statistics for all ROI‐to‐ROI connections. Figure S2 presents functional connectivity data graphically for all ROI‐to‐ROI connections.

**TABLE 2:** Results from repeated measures ANCOVA models testing the primary aim—that is, examining changes in functional connectivity from resting‐state to Montreal Imaging Stress Task (MIST) in alcohol use disorder (AUD) versus controls.

	Main effect condition		Main effect group		Condition x group interaction		Condition x age covariate		Condition x sex covariate	
	*F*	*p*_*FDR*_	*F*	*p*_*FDR*_	*F*	*p*_*FDR*_	*F*	*p*_*FDR*_	*F*	*p*_*FDR*_
Right OFC–right hippocampus	2.85	0.20	5.03	0.31	7.84	**0.048**	2.46	0.30	4.34	0.24
Left OFC–left hippocampus	0.03	0.86	3.53	0.34	0.01	0.91	0.00	0.98	0.77	0.58
Right OFC–right amygdala	0.03	0.86	0.79	0.65	10.41	**0.02**	0.46	0.75	3.75	0.23
Left OFC–left amygdala	8.35	0.07	0.06	0.89	1.96	0.48	6.75	0.08	6.66	0.16
Right OFC–right NAcc	3.57	0.20	2.92	0.34	1.16	0.49	2.80	0.30	0.21	0.74
Left OFC–left NAcc	0.29	0.72	2.60	0.34	1.69	0.48	1.20	0.48	0.20	0.74
vmPFC–right hippocampus	4.09	0.20	1.39	0.49	0.89	0.53	0.00	0.98	1.45	0.58
vmPFC–left hippocampus	3.16	0.20	1.12	0.88	1.18	0.49	0.02	0.89	0.11	0.74
vmPFC–right amygdala	0.50	0.72	0.02	0.89	0.02	0.91	3.70	0.24	1.38	0.57
vmPFC–left amygdala	1.73	0.34	0.19	0.88	2.59	0.46	0.06	0.81	0.86	0.57
vmPFC–right NAcc	0.36	0.72	1.62	0.50	0.53	0.62	1.25	0.27	0.14	0.71
vmPFC–left NAcc	5.99	0.11	0.31	0.87	0.04	0.90	8.59	0.06	0.86	0.58

*Note:* Condition (resting‐state, MIST) was the two‐level within‐subject factor, group (control, AUD) was the between‐subject factor and ROI‐to‐ROI functional connectivity (each of the 12 connections modelled separately) was the repeated measure outcome variable. Age and biological sex were included as covariates. To account for multiple comparisons (i.e., 12 ROI‐to‐ROI comparisons), we implement an FDR correction using the p.adjust function in R Statistical Software. Significant results (*pFDR* < 0.05) are in bold text.

Abbreviations: NAcc, nucleus accumbens; OFC, orbitofrontal cortex; vmPFC, ventromedial prefrontal cortex.

### 3.4 Exploratory and Sensitivity Analyses

#### 3.4.1 Exploring Correlations Between Subjective Anxiety/Distress and Functional Connectivity

There were no significant correlations between changes in subjective anxiety or distress and changes in functional connectivity between the right OFC–right amygdala and right OFC–right hippocampus, in either the AUD or control group (all *p*'s > 0.47; see Figure S1).

#### 3.4.2 Exploring Hemispheric Laterization

There was a significant hemisphere x group x condition interaction on OFC–amygdala functional connectivity (*F*_[1, 178]_ = 7.92, *p* = 0.005). Stratifying by hemisphere indicated a significant group x condition interaction in the right but not the left hemisphere (statistics presented in Table 2), providing support for right‐lateralized effects in OFC–amygdala functional connectivity. There was no significant hemisphere x group x condition interaction on OFC–hippocampus functional connectivity (*F*_[1, 178]_ = 2.36, *p* = 0.13). However, there was a significant main effect of hemisphere (*F*_[1, 178]_ = 12.17, *p* < 0.001), indicating overall differences in OFC–hippocampus functional connectivity between right and left hemispheres. Full model results are detailed in Table S2.

#### 3.4.3 Temporal Signal‐To‐Noise Ratio (tSNR) Sensitivity Analyses

Mean tSNR values for OFC, amygdala and hippocampus ROIs are reported in Table S3. tSNR maps of the OFC overlayed on an anatomical image are shown in Figure S3. tSNR values fell within acceptable ranges for these regions, ranging from 62.62 to 73.37 [61]. Although the groups did not differ in their OFC, amygdala or hippocampus tSNR during the resting‐state scan, the AUD group had significantly lower OFC (*p* = 0.002), amygdala (*p* = 0.004) and hippocampus (*p* = 0.004) tSNR than the control group during the MIST. To examine the potential influence of this discrepancy, we conducted a sensitivity analysis controlling for tSNR during the MIST. Primary findings remain significant when controlling for tSNR (group x condition on right OFC–right amygdala: *F*_[1, 41]_ = 4.18, *p* = 0.047; group x condition on right OFC—right hippocampus: *F*_[1, 41]_ = 5.38, *p* = 0.025).

#### 3.4.4 Cannabis Use Sensitivity Analyses

Primary findings remained significant when excluding participants who tested positive for THC prior to fMRI scan (R OFC–R amygdala: group x condition, *F*_[1, 40]_ = 10.5, *p* = 0.002; R OFC–R hippocampus: group x condition, *F*_[1, 40]_ = 6.2, *p* = 0.017).

### 3.5 Associations Between Neural Flexibility and Coping Strategies

**FIGURE 2:**
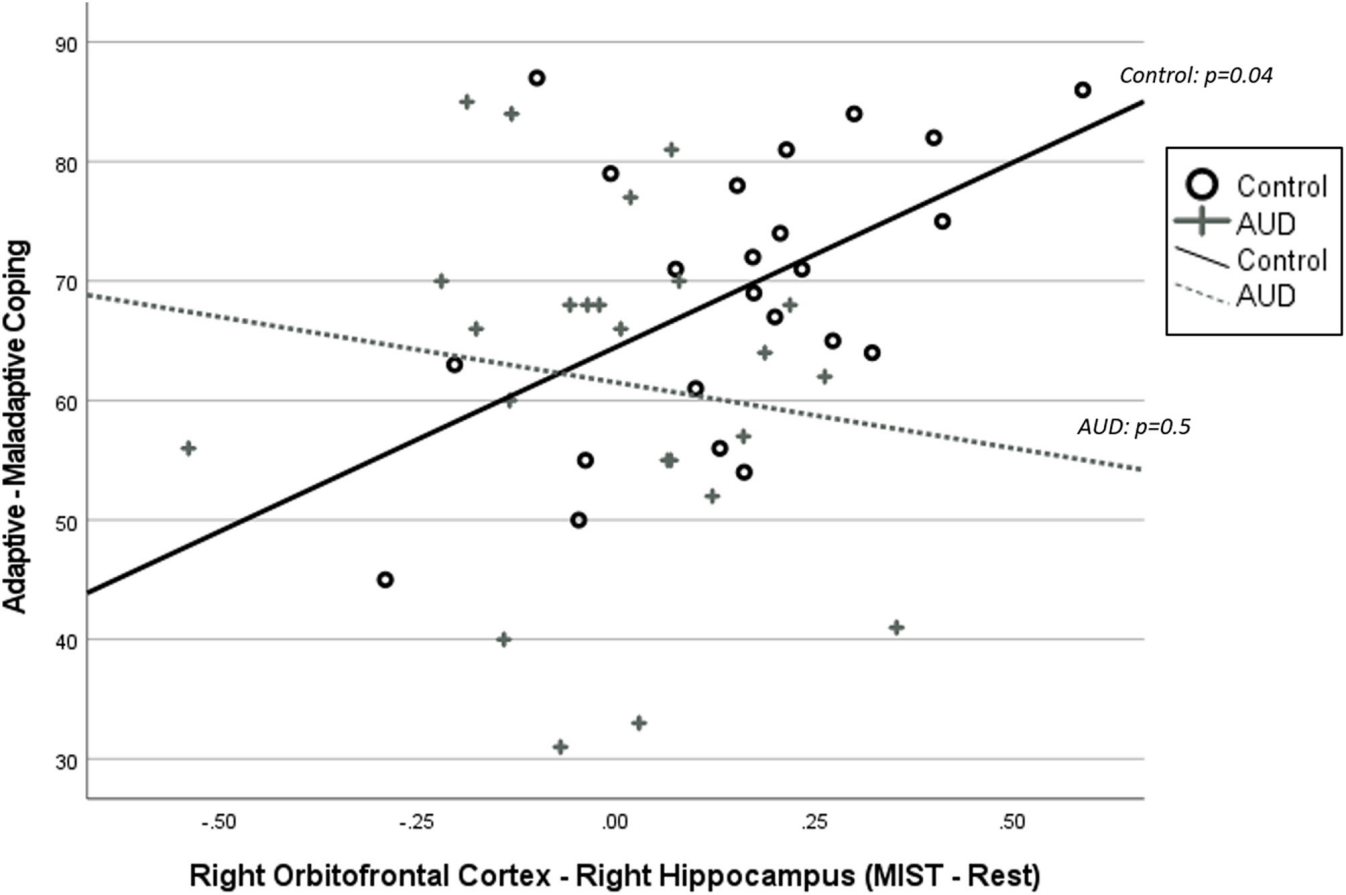
Correlation between change in right orbitofrontal cortex (OFC)–right hippocampus functional connectivity (Montreal Imaging Stress Task [MIST]—rest) and adaptive versus maladaptive (avoidant) coping score. Group moderated the relationship between right OFC–right hippocampus connectivity and coping (*p* = 0.047). Higher right OFC–right hippocampus functional connectivity from rest to MIST was associated with greater adaptive versus maladaptive coping strategies in the control group (*p* = 0.04) but not in the AUD group (*p* = 0.5).

We calculated change in right OFC–right amygdala and right OFC–right hippocampus functional connectivity (MIST minus rest). Group moderated the relationship between right OFC–right hippocampus connectivity and coping strategies (*t*_[41]_ *=* 2.05, *p* = 0.047, CI: −81.5, −0.6; Figure 2). Higher right OFC–right hippocampus functional connectivity from rest to MIST was associated with greater adaptive versus maladaptive coping strategies in the control group (*t*_[19]_ = 2.07, *p* = 0.04, CI: 0.8, 59.3) but not in the AUD group (*t*_[20]_ = −0.8, *p* = 0.45, CI: −40.2, 18.2). There was no relationship between right OFC–right amygdala connectivity and coping strategies (*t*_[41]_ = −0.4, *p* = 0.7, CI: −60.8, 42.2).

Given the group differences in resting state functional connectivity, we repeated the above moderation models with resting‐state functional connectivity as the independent variable. There was no significant group x resting‐state functional connectivity interaction on coping (right OFC–right amygdala: *t*_[41]_ = −0.62, *p* = 0.54, CI: −72.23, 38.20; right OFC–right hippocampus: *t*_[41]_ = 0.82, *p* = 0.42, CI: −29.05, 68.88). Additionally, there were no main effects of resting‐state functional connectivity on coping (right OFC–right amygdala: *t*_[41]_ = 0.47, *p* = 0.64, CI: −31.60, 50.86; right OFC–right hippocampus: *t*_[41]_ = −0.61, *p* = 0.55, CI: −46.70, 25.12).

## 4 Discussion

This study examined neural flexibility, defined as changes in functional connectivity from resting‐state to task‐based stress induction, between vPFC and limbic/striatal regions in adults with AUD versus matched controls. Consistent with our hypothesis, individuals with moderate‐to‐severe AUD exhibited neural inflexibility, or greater similarity in resting‐state and MIST functional connectivity patterns, compared to healthy controls. Specifically, the control group showed an increase in functional connectivity between right OFC–right amygdala and right OFC–right hippocampus from resting‐state to the MIST, whereas the AUD group demonstrated no such change. Moreover, in controls, *increases* in right OFC–right hippocampus functional connectivity were positively associated with more adaptive versus maladaptive coping skills. As there was no relationship between resting‐state functional connectivity and coping, this suggests that it is the change, rather than static connectivity alone, that may be behaviourally relevant. There were no associations between functional connectivity changes and coping among individuals with AUD. These findings inform our understanding of how the circuits involved in the stress response may be altered in AUD, suggesting impaired flexibility of the system in response to changing stress conditions.

Although the AUD and control groups did not differ in functional connectivity during the MIST, they differed in their functional connectivity at rest. Specifically, individuals with AUD had stronger resting‐state functional connectivity between right OFC–right amygdala and right OFC–right hippocampus compared to healthy controls. Stronger medial PFC to amygdala connectivity at rest has been associated with sustained stress system activation, as reflected by a slowed decline in cortisol following its morning peak in healthy men [34]. Furthermore, the AUD group self‐reported greater distress compared to the control group prior to their resting‐state scan. Although speculative, it is possible this pattern of resting‐state connectivity reflects heightened stress system engagement, or an allostatic state, at baseline in AUD. Persistent stress system dysregulation is thought to impair the ability to appropriately respond to additional stressors [3, 24]. Prior studies have shown that individuals with high baseline cortisol levels exhibit a blunted cortisol response to stress [5], a pattern that has been linked to enhanced drug motivation and self‐administration in individuals with a substance use disorder [5, 62, 63, 64]. Indeed, resting‐state functional connectivity is thought to provide a baseline from which neural networks dynamically reconfigure to meet task‐specific demands [35, 65, 66, 67, 68]. Based on this literature, we speculate that elevated engagement of these OFC‐limbic systems at rest may reflect an allostatic state that limits the system's capacity to flexibly respond to acute stress. Future studies are needed to directly test this hypothesis and implement methodology that could help to disentangle causality. For example, reverse translational animal studies using circuit‐specific manipulations (e.g., optogenetics) could provide causal evidence for whether resting‐state connectivity impacts flexible stress responses.

Interestingly, the observed findings were lateralized to the right hemisphere, with no significant effects detected in the left hemisphere. Exploratory analyses testing whether group by condition interactions statistically differed by hemisphere provide further support for this lateralization, specifically for OFC–amygdala functional connectivity. This pattern is consistent with prior research implicating right‐lateralized frontolimbic circuits in salience detection and emotional reactivity/processing [13, 69]. Although lateralization of brain function during resting‐state or stress conditions has not been extensively studied in AUD, structural MRI studies in AUD have reported greater morphological abnormalities in the right hemisphere, whereas cue‐induced craving fMRI paradigms have shown left‐lateralized functional responses [70]. These findings highlight the need for future research to systematically examine hemispheric asymmetries in AUD and evaluate their potential clinical significance.

More broadly, our findings further underscore the importance of neural flexibility in adaptive stress coping. Among healthy controls, increased right OFC–right hippocampus functional connectivity from resting state to acute stress was associated with more adaptive versus maladaptive stress coping strategies. This aligns with Sinha et al.'s study, which found that dynamic increases in vmPFC activation during stress (compared to neutral conditions) signalled active, resilient coping in healthy adults [13]. Specifically, the study observed a pattern of initial vmPFC hypoactivity followed by vmPFC mobilization—or increased activation—in response to acute stress. Flexibility of vmPFC responses to stress was associated with individual differences in coping behaviour. Functional mobilization of the vmPFC positively related to active coping, whereas lack of vmPFC mobilization was associated with maladaptive coping behaviours, including higher frequency of binge drinking. Similarly, another study found that greater flexibility, reflected by more time‐varying interactions between rostral anterior cingulate cortex (ACC) and amygdala at rest, was associated with stress resilience in women with a history of early life stress [71]. Although the aforementioned studies took a different approach—examining flexibility within a stress task or resting state, respectively—findings converge to suggest that flexibility within the vPFC systems is crucial for supporting adaptive stress responses. Cognitive neuroscience research also supports that flexible reconfiguration of brain networks as a function of state underlies adaptive cognitive control [37, 41, 42].

Our findings of neural inflexibility in AUD align with research suggesting inflexible state‐dependent connectivity changes in mood disorders. For example, Ho et al. [36] found that adolescents with MDD exhibited more similar dorsal ACC functional connectivity patterns during resting‐state and response inhibition task performance. Similarly, another study found that adolescents with MDD showed inflexibly elevated functional connectivity within the default mode network, specifically between the subcallosal cingulate cortex and posterior cingulate cortex, across an emotional identification task and resting‐state, compared to healthy peers [43]. Together, these findings suggest atypically inflexible PFC‐based functional connectivity may be a transdiagnostic feature of psychopathology, potentially contributing to maladaptive responses to changing cognitive and emotional demands.

Several study strengths and limitations should be considered when interpreting results. Strengths include well‐matched groups with racial and ethnical diversity and a balanced representation of men and women. Additionally, this study applied a methodologically sound approach grounded in cognitive neuroscience and mood disorder research [35, 36, 37, 38, 39, 40], extending its application to a clinical AUD sample. Study limitations include a relatively small sample size and cross‐sectional design. Therefore, we were not powered to investigate interactions with sex, a critical biological variable that influences the stress response in AUD [54, 72, 73]. Additionally, participants were informed they would be completing math problems (during the MIST) but were unaware that the task was designed to act as an acute stressor. Given the differences in neural sensitivity to predictable versus uncertain stress in AUD [74], further research is needed to determine how connectivity patterns vary with stress predictability. Another important consideration is that the resting‐state scan always occurred before the stress task. Therefore, we cannot rule out the possibility that the observed changes reflect time‐dependent effects, or the cognitive nature of the task (i.e., arithmetic), rather than stress per se. Future studies using counterbalanced designs, or stress tasks with limited cognitive load, could help to disentangle these potential confounds. Additionally, future studies would benefit from collecting biological markers of the stress response (e.g., cortisol), which could help inform the interpretation of functional connectivity findings. Additionally, investigating neural flexibility from rest to stress tasks involving instructed stress regulation (e.g., cognitive reappraisal [75]) could provide further insight into the systems underlying adaptive stress regulation. Lastly, we observed group differences in tSNR values during the MIST, with the AUD group exhibiting significantly lower tSNR than the control group. However, tSNR values in the AUD group remained within acceptable limits, and sensitivity analyses controlling for tSNR during the MIST remained significant. These results suggest that the observed findings are not driven by differences in signal quality; nonetheless, we acknowledge this as a methodological consideration when interpreting findings.

In summary, this study identified a pattern of neural inflexibility between the right OFC–right amygdala and right OFC–right hippocampus in individuals with moderate‐to‐severe AUD, compared to matched controls, under changing stress conditions. Functional connectivity between these regions increased from resting state to the acute stress task in the control group; however, this shift was not present in the AUD group. Although the AUD and control groups did not differ in their functional connectivity between these regions during the stress task, the groups showed significant differences at rest. Specifically, the AUD group exhibited elevated right OFC–right amygdala and right OFC–right hippocampus resting state functional connectivity. Future studies are needed to test whether this pattern of connectivity reflects persistent dysregulation within these stress response systems, or an allostatic state, that ultimately limits the system's capacity to flexibly respond to acute stress.

## Author Contributions

**Dylan E. Kirsch:** conceptualization, methodology, formal analysis, investigation, data curation, writing – originial draft, visualization. **Tiffany C. Ho:** conceptualization, methodology, writing – review and editing. **Kate M. Wassum:** supervision, writing – review and editing. **Lara A. Ray:** conceptualization, resources, writing – review and editing, supervision, funding acquisition. **Erica N. Grodin:** resources, writing – review and editing, supervision, funding acquisition.

## Ethics Statement

Study procedures were approved by the Institutional Review Board at UCLA (IRB #22‐001951).

## Consent

All participants provided written informed consent to participate.

## Conflicts of Interest

The authors declare no conflicts of interest.

## Supporting information


**Figure S1:** adb70083‐sup‐0001‐Suppl_Material.docx. **(A)** Subjective Units of Distress (SUDs) and **(B)** State Trait Anxiety Inventory (STAI) scores (estimated marginal means) before and after acute stress induction via the Montreal Imaging Stress Task (MIST). Across both groups, participants reported greater distress (SUDS; *p* = 0.04) and anxiety (STAI; *p* = 0.03) after the MIST, compared to before. There was a main effect of group on distress (*p* = 0.02) such that the AUD group reported greater distress both before and after the MIST.
**Figure S2:** Functional connectivity between all ROI‐to‐ROI pairs during resting state and Montreal Imaging Stress Task (MIST). The y‐axis displays the estimated marginal means (from primary ANOVA models). Model statistics are details **Table 2**.
**Table S1:** Partial correlations between changes in functional connectivity and changes in subjective stress.
**Table S2:** Exploratory Lateralization Analyses: Orbitofrontal Cortex—Amygdala Functional Connectivity.
**Table S3:** Average temporal signal‐to‐noise ratio (tSNR) values for the amygdala, hippocampus, and orbitofrontal cortex (OFC) during the resting‐state and MIST scans.
**Figure S3:** Temporal signal‐to‐noise ratio (tSNR) maps of the orbitofrontal cortex (OFC) overlaid on an anatomical image.

## Data Availability

The data that support the findings of this study are available from the corresponding author upon reasonable request.
