# Supplementary material for: Inflexible Orbitofrontal Cortex Functional Connectivity From Rest to Acute Stress in Alcohol Use Disorder
Source: Addict Biol. 2025 Aug 17;30(8):e70083. doi: 10.1111/adb.70083 (PMC12358688; doi:10.1111/adb.70083)

**Supplemental Material**

**Supplemental Figure 1.**

**
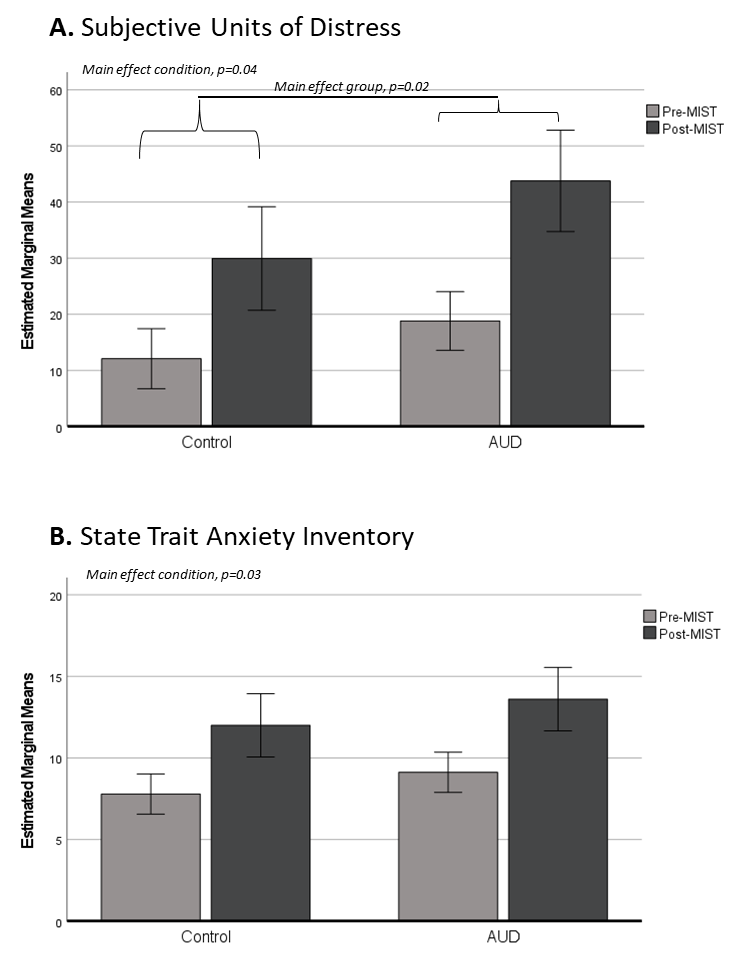
**

**(A)** Subjective Units of Distress (SUDs) and **(B)** State Trait Anxiety Inventory (STAI) scores (estimated marginal means) before and after acute stress induction via the Montreal Imaging Stress Task (MIST). Across both groups, participants reported greater distress (SUDS; p=0.04) and anxiety (STAI; p=0.03) after the MIST, compared to before. There was a main effect of group on distress (p=0.02) such that the AUD group reported greater distress both before and after the MIST.

**Supplemental Figure 2.**

**
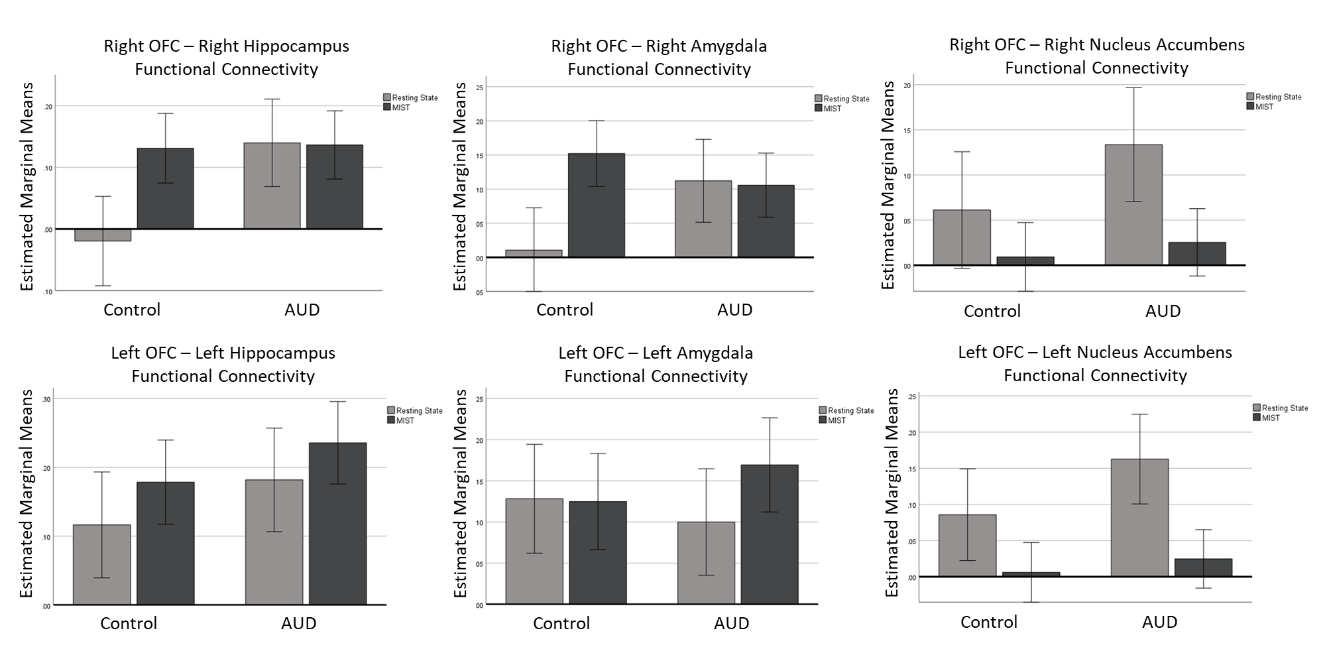
**

**
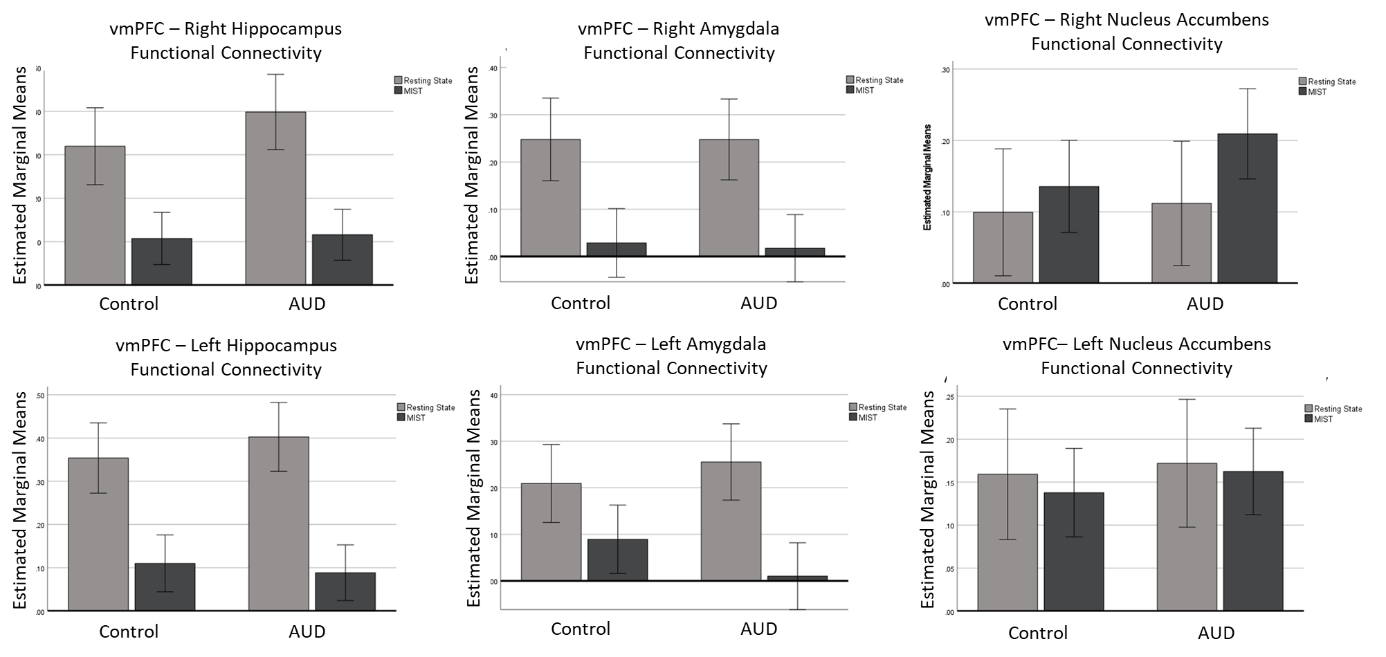
**

Functional connectivity between all ROI-to-ROI pairs during resting state and Montreal Imaging Stress Task (MIST). The y-axis displays the estimated marginal means (from primary ANOVA models). Model statistics are details **Table 2**.

**Supplemental Table 1.**

Partial correlations between changes in functional connectivity and changes in subjective stress.

|  | STAI Change (MIST – Rest) | SUDS Change (MIST – Rest) |
| --- | --- | --- |
| **AUD GROUP** | | |
| R OFC – R Hippocampus FC Change (MIST – Rest) | r=-0.019  p=0.935 | r=0.033  p=0.88 |
| R OFC – R Amygdala FC Change (MIST – Rest) | r=0.072  p=0.757 | r=0.118  p=0.612 |
| **CONTROL GROUP** | | |
| R OFC – R Hippocampus FC Change (MIST – Rest) | r=0.099  p=0.669 | r=0.168  p=0.466 |
| R OFC – R Amygdala FC Change (MIST – Rest) | r=0.018  p=0.938 | r=-0.019  p=0.934 |

**Supplemental Table 2.**

Exploratory Lateralization Analyses: Orbitofrontal Cortex – Amygdala Functional Connectivity

| **Source** | **Type III Sum of Squares** | **df** | **Mean Square** | **F** | **Sig.** |
| --- | --- | --- | --- | --- | --- |
| Corrected Model | .795^a^ | 9 | 0.088 | 4.23 | **<.001** |
| Intercept | 0.141 | 1 | 0.141 | 6.774 | **0.01** |
| Sex | 0.039 | 1 | 0.039 | 1.863 | 0.174 |
| Age | 0.017 | 1 | 0.017 | 0.83 | 0.363 |
| Hemisphere | 0.201 | 1 | 0.201 | 9.635 | **0.002** |
| Group | 0.021 | 1 | 0.021 | 0.996 | 0.32 |
| Condition | 0.303 | 1 | 0.303 | 14.495 | **<.001** |
| Hemisphere * Group | 0.003 | 1 | 0.003 | 0.144 | 0.704 |
| Hemisphere * Condition | 0.008 | 1 | 0.008 | 0.361 | 0.549 |
| Group * Condition | 0.013 | 1 | 0.013 | 0.601 | 0.439 |
| Hemisphere * Group * Condition | 0.165 | 1 | 0.165 | 7.916 | **0.005** |
| Error | 3.717 | 178 | 0.021 |  |  |
| Total | 7.597 | 188 |  |  |  |
| Corrected Total | 4.511 | 187 |  |  |  |
| *^a^ R Squared = .176 (Adjusted R Squared = .135)* | | | | | |

Exploratory Lateralization Analyses: Orbitofrontal Cortex – Hippocampus Functional Connectivity

| **Source** | **Type III Sum of Squares** | **df** | **Mean Square** | **F** | **Sig.** |
| --- | --- | --- | --- | --- | --- |
| Corrected Model | .983^a^ | 9 | 0.109 | 4.294 | **<.001** |
| Intercept | 0.014 | 1 | 0.014 | 0.533 | 0.466 |
| Sex | 0.016 | 1 | 0.016 | 0.647 | 0.422 |
| Age | 0.078 | 1 | 0.078 | 3.063 | 0.082 |
| Hemisphere | 0.31 | 1 | 0.31 | 12.168 | **<.001** |
| Group | 0.242 | 1 | 0.242 | 9.505 | **0.002** |
| Condition | 0.203 | 1 | 0.203 | 7.989 | **0.005** |
| Hemisphere * Group | 0.006 | 1 | 0.006 | 0.223 | 0.637 |
| Hemisphere * Condition | 0.003 | 1 | 0.003 | 0.113 | 0.737 |
| Group * Condition | 0.077 | 1 | 0.077 | 3.03 | 0.083 |
| Hemisphere * Group * Condition | 0.06 | 1 | 0.06 | 2.355 | 0.127 |
| Error | 4.529 | 178 | 0.025 |  |  |
| Total | 9.107 | 188 |  |  |  |
| Corrected Total | 5.512 | 187 |  |  |  |
| *^a^ R Squared = .178 (Adjusted R Squared = .137)* | | | | | |

**Supplemental Table 3.**

Average temporal signal-to-noise ratio (tSNR) values for the amygdala, hippocampus, and orbitofrontal cortex (OFC) during the resting-state and MIST scans.

|  | **Total** | | **Control** | | **AUD** | | **Control vs. AUD** |
| --- | --- | --- | --- | --- | --- | --- | --- |
|  | **Mean** | **SD** | **Mean** | **SD** | **Mean** | **SD** | **p-value** |
| **Rest: Amygdala tSNR** | 65.95 | 10.27 | 66.30 | 9.03 | 65.60 | 11.52 | *0.820* |
| **Rest: Hippocampus tSNR** | 73.37 | 10.68 | 74.83 | 10.34 | 71.97 | 11.03 | *0.360* |
| **Rest: OFC tSNR** | 68.45 | 14.01 | 71.43 | 13.46 | 65.60 | 14.21 | *0.160* |
| **MIST: Amygdala tSNR** | 63.60 | 12.30 | 68.69 | 9.28 | 58.73 | 13.01 | ***0.004*** |
| **MIST: Hippocampus tSNR** | 67.84 | 12.50 | 73.02 | 10.50 | 62.88 | 12.44 | ***0.004*** |
| **MIST: OFC tSNR** | 62.62 | 14.02 | 68.86 | 9.75 | 56.64 | 15.03 | ***0.002*** |

**Supplemental Figure 3.**

Temporal signal-to-noise ratio (tSNR) maps of the orbitofrontal cortex (OFC) overlaid on an anatomical image.


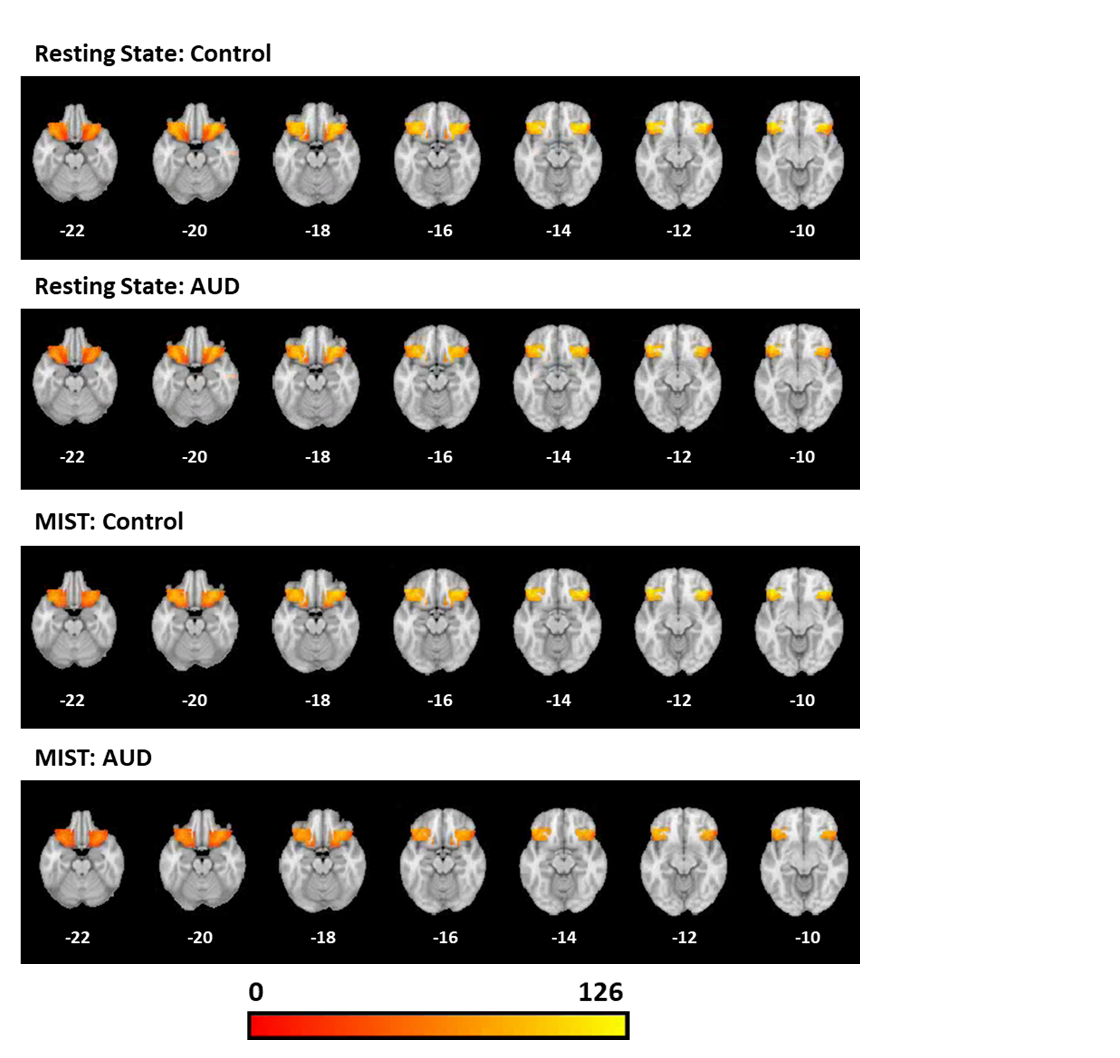

Supplement: Supplementary file 1 — Figure S1: adb70083‐sup‐0001‐Suppl_Material.docx. (A) Subjective Units of Distress (SUDs) and (B) State Trait Anxiety Inventory (STAI) scores (estimated marginal means) before and after acute stress induction via the Montreal Imaging Stress Task (MIST). Across both groups, participants reported greater distress (SUDS; p = 0.04) and anxiety (STAI; p = 0.03) after the MIST, compared to before. There was a main effect of group on distress (p = 0.02) such that the AUD group reported greater distress both before and after the MIST. Figure S2: Functional connectivity between all ROI‐to‐ROI pairs during resting state and Montreal Imaging Stress Task (MIST). The y‐axis displays the estimated marginal means (from primary ANOVA models). Model statistics are details Table 2. Table S1: Partial correlations between changes in functional connectivity and changes in subjective stress. Table S2: Exploratory Lateralization Analyses: Orbitofrontal Cortex—Amygdala Functional Connectivity. Table S3: Average temporal signal‐to‐noise ratio (tSNR) values for the amygdala, hippocampus, and orbitofrontal cortex (OFC) during the resting‐state and MIST scans. Figure S3: Temporal signal‐to‐noise ratio (tSNR) maps of the orbitofrontal cortex (OFC) overlaid on an anatomical image. [file ADB-30-e70083-s001.docx]
